# Usutu Virus Infection of Embryonated Chicken Eggs and a Chicken Embryo-Derived Primary Cell Line

**DOI:** 10.3390/v12050531

**Published:** 2020-05-12

**Authors:** Emna Benzarti, José Rivas, Michaël Sarlet, Mathieu Franssen, Nassim Moula, Giovanni Savini, Alessio Lorusso, Daniel Desmecht, Mutien-Marie Garigliany

**Affiliations:** 1 Fundamental and Applied Research for Animals & Health (FARAH), Faculty of Veterinary Medicine, University of Liège, Sart Tilman B43, B-4000 Liège, Belgium; ebenzarti@uliege.be (E.B.); jfrivast@gmail.com (J.R.); Michael.Sarlet@uliege.be (M.S.); mfranssen@uliege.be (M.F.); Nassim.Moula@uliege.be (N.M.); daniel.desmecht@uliege.be (D.D.); 2 OIE Reference Centre for West Nile Disease, Istituto Zooprofilattico Sperimentale “G. Caporale”, 46100 Teramo, Italy; g.savini@izs.it (G.S.); a.lorusso@izs.it (A.L.)

**Keywords:** flavivirus, chicken embryo, model, Usutu virus, chorioallantoic membrane, primary culture, replication

## Abstract

Usutu virus (USUV) is a mosquito-borne flavivirus, closely related to the West Nile virus (WNV). Similar to WNV, USUV may cause infections in humans, with occasional, but sometimes severe, neurological complications. Further, USUV can be highly pathogenic in wild and captive birds and its circulation in Europe has given rise to substantial avian death. Adequate study models of this virus are still lacking but are critically needed to understand its pathogenesis and virulence spectrum. The chicken embryo is a low-cost, easy-to-manipulate and ethically acceptable model that closely reflects mammalian fetal development and allows immune response investigations, drug screening, and high-throughput virus production for vaccine development. While former studies suggested that this model was refractory to USUV infection, we unexpectedly found that high doses of four phylogenetically distinct USUV strains caused embryonic lethality. By employing immunohistochemistry and quantitative reverse transcriptase-polymerase chain reaction, we demonstrated that USUV was widely distributed in embryonic tissues, including the brain, retina, and feather follicles. We then successfully developed a primary cell line from the chorioallantoic membrane that was permissive to the virus without the need for viral adaptation. We believe the future use of these models would foster a significant understanding of USUV-induced neuropathogenesis and immune response and allow the future development of drugs and vaccines against USUV.

## 1. Introduction

Usutu virus (USUV) is a zoonotic arbovirus related to Japanese encephalitis (JEV) and West Nile (WNV) viruses (genus *Flavivirus*, family *Flaviviridae*) [1]. Initially restricted to Africa, it emerged in Europe in 1996 and managed to establish an endemic mosquito–bird life cycle and to co-circulate with WNV in many European countries [2,3]. Further, its rapid geographic spread across Europe led to a noteworthy recrudescence of infections in birds, recorded in over 96 species from 36 families [4,5,6], as well as substantial avian mortalities, especially in Eurasian blackbirds (*Turdus merula*) [7,8]. 

As for WNV, most human USUV infections are asymptomatic. In total, more than 80 cases of subclinical infections were described in blood donors or persons with risk of exposure in Italy, Serbia, the Netherlands, and Germany during WNV surveillance surveys, until now [9,10,11,12,13]. Seroprevalence studies showed that humans are more exposed to USUV than to WNV in northern Italy, where both viruses co-circulate [11,12]. Rare cases with mild flu-like illness or neuroinvasive disease may, however, occur due to USUV infection. Between 2009 and 2018, more than 32 USUV symptomatic infections were reported in humans [14,15,16], including cases with meningoencephalitis [14,15,17,18,19]. Signs like headache, fever, nuchal rigidity, hand tremor, hyperreflexia [19], and facial paralysis [20] were described. Whether these cases of infection represent an emerging part of the iceberg and whether the incidence of USUV diseases may be underdiagnosed is still uncertain [19]. In fact, USUV might be misdiagnosed as WNV when the signs are quite similar and the diagnosis is based only on antibody detection due to cross-reactivity [21]. Besides, given the similarities in the biological, ecological, and epidemiological properties with WNV, USUV has the potential to be introduced into North America in the future [22]. Further, the ability of RNA viruses to mutate rapidly and adapt to their hosts is well known [23] and USUV could emerge as a major risk for public health in the forthcoming years or decades. Thus, there is an urgent need for research work into this virus using appropriate experimental models. 

Embryonated chicken eggs (ECE) are considered a valuable, low-cost and ethically acceptable model for human and veterinary [24,25,26] vaccine manufacturing and for the amplification and study of important flaviviruses for humans, such as Zika virus (ZIKV) [27,28] and Yellow Fever virus [29,30]. Prior studies suggested that ECE were resistant to USUV infection [31,32] and did not amplify the virus from positive dead bird samples in Italy, unlike Vero cells used in the same study [8]. In contrast, chicken embryos were successfully infected with other mosquito-borne flaviviruses known to be pathogenic for birds, such as WNV [33] and Tembusu virus [34]. Hence, the finding that ECE were refractory to USUV infection was unexpected, as birds are known to be the most susceptible hosts for USUV [8,31]. Previous studies using the MR766 ZIKV strain showed that primary embryonic chicken cells were not susceptible to infection [35], while recent studies demonstrated that the DF-1 chicken fibroblast cell line [36] and chicken embryos were susceptible to infection by currently-circulating ZIKV strains [27,28]. Therefore, to characterize the pathogenicity *in ovo* of contemporary USUV strains [37] and to research for a useful avian model for the study of this epornitic virus, we inoculated ECE with high doses of a USUV strain that we isolated during an avian outbreak in Belgium in 2017 [37]. Unexpectedly, this USUV strain replicated in the allantoic fluids (AFs) and embryonic tissues and induced dose-dependent mortality rates in chicken embryos. We subsequently infected ECE with three other strains, each representative of a different lineage of USUV (Africa 3 and Europe 1 and 2). In parallel, as we identified the chorioallantoic membrane (CAM) as a predilection site for viral replication, we isolated cells from this tissue and assessed the growth kinetics of USUV strains using this in vitro model. 

## 2. Materials and Methods 

### 2.1. Viruses and Embryonated Chicken Eggs

Size-matched fertile chicken eggs (Lohmann Brown strain) were obtained from De Biest (Kruishoutem, Belgium). USU-BE-Seraing/2017 (*Genbank*: MK230892, lineage: Europe 3, passage 5) and USU-BE-Grivegnee/2017 (*Genbank:* MK230891, lineage: Africa 3, passage 5) strains were isolated in our laboratory from dead Eurasian blackbird (*Turdus merula*) tissues collected in Belgium in 2017 [37]. USUV strain Vienna 2001 (*Genbank*: AY453411, lineage: Europe 1, passage 17) was isolated from a dead blackbird in 2001 in Austria and UR-10-Tm strain (*GenBank*: KX555624, lineage: Europe 2, passage 5) was isolated from a dead blackbird in 2010 in Italy. Viruses were amplified in African Green Monkey Vero cells (ATCC CRL-1586 VERO C1008) using Dulbecco’s Minimum Essential Medium (DMEM, Lonza, Verviers, Belgium) cell culture medium supplemented with 1% penicillin/streptomycin. The culture supernatants were titrated by the 50% tissue culture infective dose (TCID_50_) technique and kept at −80 °C until use. 

### 2.2. In Ovo Characterization of USU-BE-Seraing/2017

For the survival study, three different doses of USU-BE-Seraing/2017 strain (10^4^, 10^5^, or 10^6^ TCID_50_ dispersed in 100 µL of infected Vero cell culture supernatant diluted using DMEM) were each injected into nine 10-day-old ECE via the allantoic route. The eggs were subsequently incubated together with nine mock-infected controls at 37.5 °C and 55% relative air humidity. All eggs were daily checked by candling for embryonic vitality during 6 days post-infection (dpi). After the identification of embryonic death, the corresponding allantoic liquid was harvested and samples from the CAM, liver, skeletal muscle, heart, and brain were collected and examined by histology and immunohistochemistry (IHC) as in [38]. Virus isolation in 24-well plates containing a confluent monolayer of Vero cells was attempted from the allantoic fluid and liver tissues of each dead embryo [8].

To study the time-course of infection using the USU-BE-Seraing/2017 strain, a set of 62 ECE in the tenth day of development was incubated at 37.5 °C following allantoic cavity inoculation with 100 µL of infected Vero cell culture supernatant yielding an infectious dose of 10^5^ TCID_50_. As negative controls, 30 eggs were injected via the allantoic route with 100 µL of virus-free DMEM. Over 5 dpi, dead embryos were opened and AFs were harvested to quantify RNA loads by RT-qPCR. In parallel, eight live infected and six uninfected age-matched embryos were randomly selected each day for euthanasia by decapitation. AF samples (200 µL) from the infected embryos were harvested to assess viral replication by RT-qPCR. Tissue samples from the CAMs, livers, hearts, and brains of five embryos were collected for RT-qPCR, histology, and IHC examination [38]. Viral RNA copies (VRC) in each tissue were calculated using a standard curve, which was constructed as described in [39]. The remaining embryos (three infected and one uninfected) were dissected as follows: for each embryo, the head, whole wings, and whole legs were separated from the trunk, which was transversely sectioned. All fragments were then immersed in 10% neutral buffered formalin for histopathological examination. On day 5 post-infection (pi), embryos were weighted to evaluate the impact of USUV infection on their growth. 

### 2.3. Virulence of Other USUV Strains In Ovo 

To compare the virulence of USU-BE-Seraing/2017 strain *in ovo* with that of other USUV strains, three different doses of USU-BE-Grivegnee/2017, Vienna 2001, and UR-10-Tm strains (10^4^, 10^5^, or 10^6^ TCID_50_ dispersed in 100 µL of infected Vero cell culture supernatants diluted using DMEM) were each injected into nine 10-day-old ECE via the allantoic route. The ECE were kept at a controlled temperature of 37.5 °C and 55% relative air humidity. The eggs were then candled daily over 6 days. Upon detection of embryo mortality, the corresponding egg was opened and processed as previously described.

### 2.4. Preparation of Primary Chorioallantoic Membrane Cells

Primary chicken CAM cells were prepared from one 10-day-old embryo as follows: the CAM was carefully dissected, washed with phosphate-buffered saline (PBS, Gibco), and then minced into small fragments using a sterile blade. Next, the tissue was digested with 5 mL of TrypLE Select solution (Gibco, Life Technologies) at 37 °C for 10 min in a 15 mL sterile tube. The trypsinate was homogenized in the middle of the reaction by vigorous agitation of the tube. Digestion was stopped by adding 10 mL of DMEM, supplemented with 10% fetal bovine serum and 1% penicillin/streptomycin. After centrifugation at 400× *g* for 5 min, the supernatant was removed and CAM cells were re-suspended in 10 mL of the same cell culture medium. Next, the cells were filtered through a 100 µm filter and 10^7^ cells were distributed in a 25 cm^2^ flask. The cells were subsequently incubated at 37 °C with 5% CO_2_. The culture medium was renewed every three days and confluence was obtained within 7 days. The cells were passaged in a 75 cm^2^ flask; every 10 days, subcultures were obtained with a split ratio of 1:3.

### 2.5. Characterization of USUV Strains Growth Kinetics in Chorioallantoic Membrane Cells

Chicken CAM cells (passage 4) were seeded in 24-well culture plates to a confluence of 80%. The four USUV strains were diluted in DMEM supplemented with 1% penicillin/streptomycin to three different multiplicities of infection (MOI, 0.1, 0.01, and 0.001). Then, cells were rinsed once with PBS and each inoculum was added to 3 wells (1 mL per well). After 4 h of incubation at 37 °C, the inoculums were removed and the cells were washed with PBS. Fresh DMEM supplemented with 1% penicillin/streptomycin were added to each well (2 mL per well) and the cells were incubated at 37 °C and 5% CO_2_ for the duration of the experiment. Mock-infected CAM cells incubated with an uninfected Vero cell culture supernatant were used as controls. For 6 days, 200 µL of supernatant was harvested daily from each well and held at −80 °C in cryotubes for viral absolute quantification by RT-qPCR, as previously described. Cell monolayers were visually controlled for the presence of cytopathic effects (CPE). By the end of the experiment, cells were rinsed with PBS, fixed with 1 mL of 4% paraformaldehyde and subsequently stained by IHC as in [37], but without the antigen retrieval step.

### 2.6. Statistical Analyses

Survival curves were plotted and compared using the log-rank and Gehan-Breslow Wilcoxon tests (GraphPad Software, La Jolla, CA, USA).

To compare the RNA load per organ per day of infection, the Statistical Analysis System (SAS) Univariate procedure was used to test the normality of the data. Logarithmic transformation was performed to normalize the distribution of the data, which was revealed as nonparametric. The general linear model (Proc GLM, SAS 2001) was used to test the effects of the day, organ, or strain and day-organ interaction on the studied variables. The same procedure was used to compare viral load per strain per MOI in CAM cells. The comparison between the infected embryos weights with those of age-matched uninfected ones was performed by analyses of variance (ANOVA). The GLM was used to compare the viral RNA loads in the AFs of infected euthanized embryos per day of infection. All tests used in the previous analyses were implemented in SAS (SAS Institute Inc., Cary, NC, USA). A *p* < 0.05 was considered statistically significant.

All the data imputed in GraphPad and SAS are provided in the Supplementary Materials.

## 3. Results

### 3.1. In Ovo Characterization of USUV USU-BE-Seraing/2017

#### 3.1.1. Survival Study

**Figure 1:**
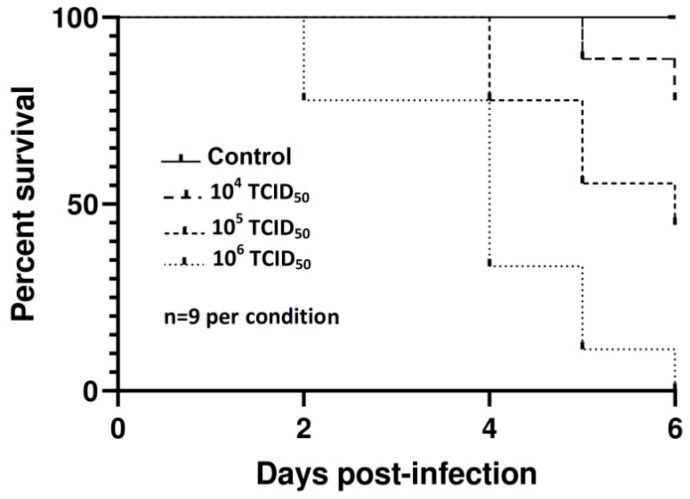
Kaplan–Meier survival curves for chicken embryos inoculated with three different doses of USU-BE-Seraing/2017 strain using the allantoic route.

Kaplan–Meier survival curves (Figure 1) showed significant differences in mortalities according to the dose by both the log-rank (Mantel-Cox) (*χ*^2^ = 16.9, *p* = 0.0002) and the Gehan-Breslow Wilcoxon tests (*χ*^2^ = 16.03, *p* = 0.0003) plotted in GraphPad Software. Mock-inoculated embryos remained alive until the end of the experiment. 

**Figure 2:**
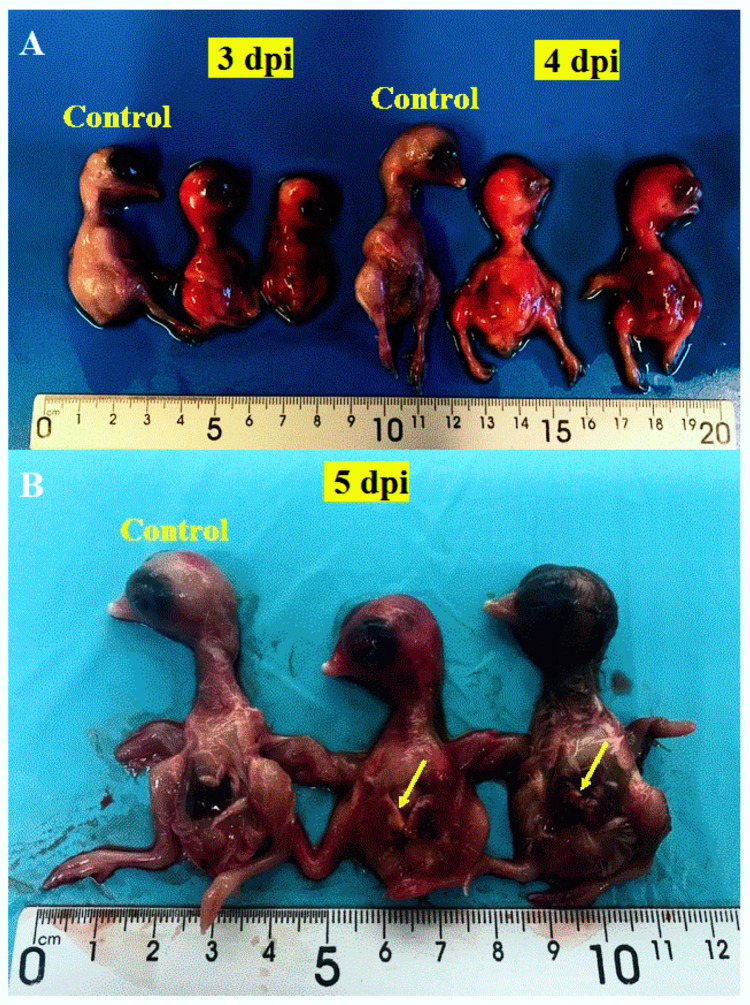
Chicken embryos after infection with USU-BE-Seraing/2017 strain using the allantoic route. (**A**) The infected chicken embryos showed cutaneous hemorrhage compared with the non-infected controls. (**B**) Unlike the non-infected embryo, the infected embryos (in the middle and on the right of the picture) died and showed cutaneous hemorrhage and pallor in the liver.

The infected dead embryos were hemorrhagic and severely swollen with edema (Figure 2). 

**Figure 3:**
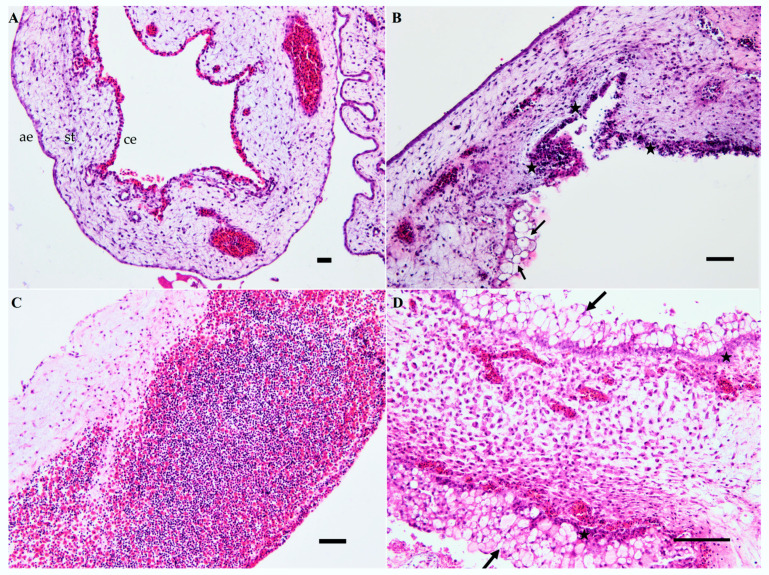
Chorioallantoic membrane from chicken embryos inoculated with the USU-BE-Seraing/2017 strain via the allantoic route. (**A**) Negative control two days after mock inoculation; (**B**) diffuse necrosis in the chorionic layer indicated by cell vacuolization (arrows) and massive nuclear fragmentation (stars) at two days post-infection (dpi); (**C**) massive infiltration of lymphocytes and heterophils in the stroma on day 5 post-infection; (**D**) Severe degeneration with vacuolization (arrows) and necrosis (stars) of cells in both epithelial layers (5 dpi). Abbreviations: ae, allantoic epithelium; ce, chorionic epithelium; st, stroma. Hematoxylin and eosin stain. Scale bars = 50 µm.

Microscopically, the most relevant feature in all of the eggs was multifocal to diffuse areas of degeneration and necrosis in the CAM, with moderate to massive infiltration of heterophils and lymphocytes (Figure 3). Most slides showed absent or severely autolytic brain tissue. 

**Figure 4:**
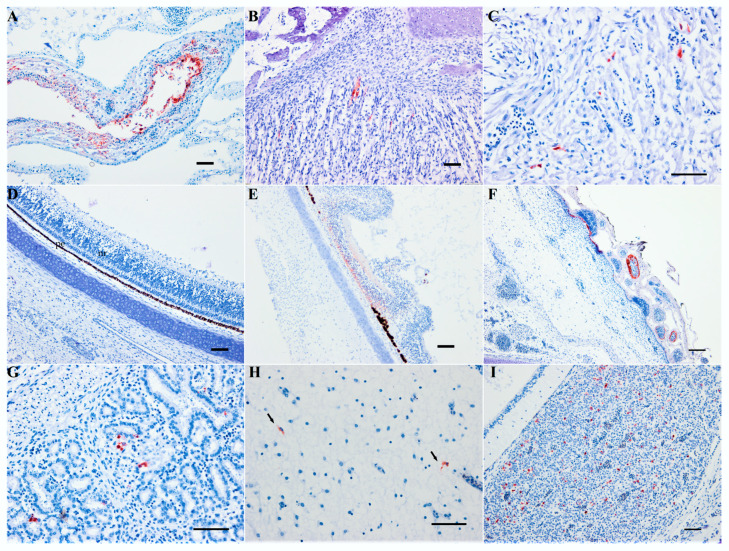
Immunohistochemical staining of Usutu virus antigens and chicken embryos. (**A**) Chorioallantoic membrane (CAM) on day 3 post-infection (pi); (**B**) skeletal muscle on day 3 pi; (**C**) heart on day 5 pi; (**D**) retina on day 3 of negative control; (**E**) retina on day 3 pi, degeneration of the neuronal layer with focal loss of the pigmented epithelium; (**F**) epidermis and feather follicle pulp on day 5 pi; (**G**) intestine, on day 5 pi; (**H**) brain on day 5 pi, UR-10-Tm strain; (**I**) pituitary gland on day 6 pi, USU-BE-Grivegnee/2017 strain. Mayer hematoxylin counterstain. Scale bars = 50 µm.

IHC revealed abundant USUV antigen in the CAM (epithelial and mesenchymal cells) and in developing myoblasts in the skeletal muscle and myocardium on day 5 pi (Figure 4A–D). A few hepatocytes were positive in a dead embryo on day 3 pi (not shown). 

Infectious viruses were successfully isolated on Vero cell cultures from the AFs and liver tissues of all infected dead embryos.

#### 3.1.2. Course of Infection

**Figure 5:**
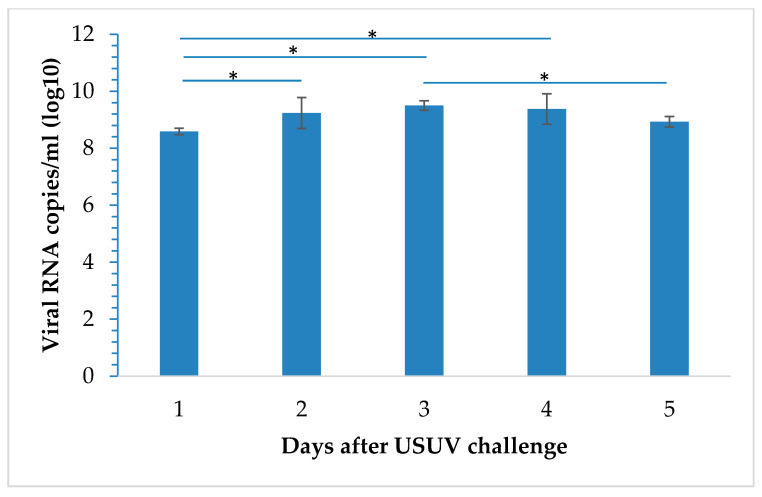
Viral RNA loads in the allantoic fluids from embryonated chicken eggs infected with USU-BE-Seraing/2017 strain at a dose of 10^5^ 50% tissue culture infective dose (TCID_50_). Data are representative of five samples per day (error bars represent the standard deviations). *n* = 5 per day of infection; “*” indicates a *p*-value < 0.05.

USUV RNA was detected in the AFs of all eggs infected with the USU-BE-Seraing/2017 strain (Figure 5). RNA loads in this region significantly varied over the infection time-course (*p* = 0.0049) and peaked on day 3 pi. Likewise, significantly higher RNA loads were found in AFs from dead embryos when compared to those from infected and euthanized ones (not shown).

**Figure 6:**
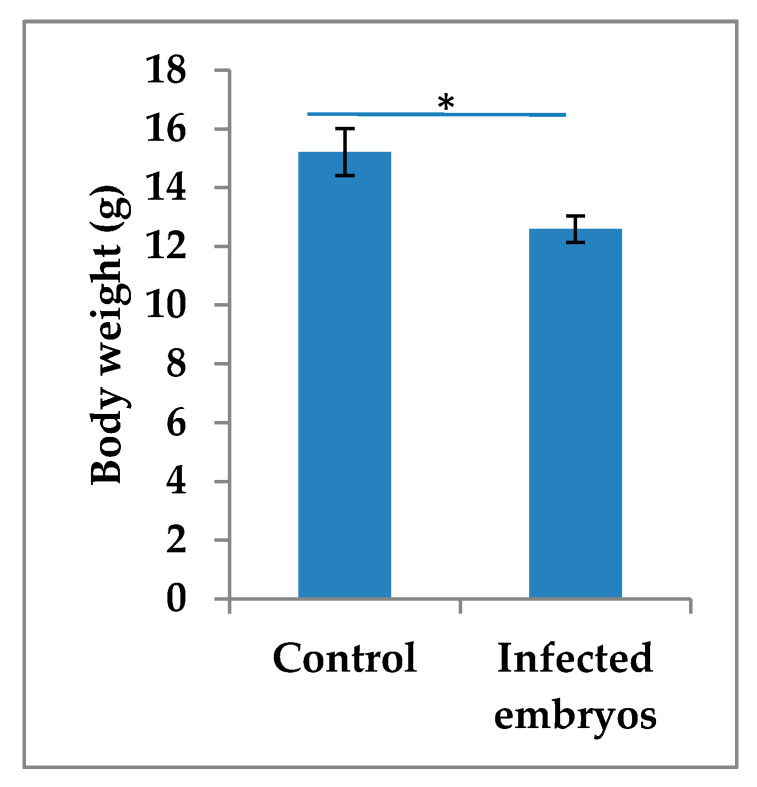
Comparison of the body weights on day 5 of the experiment between control and infected chicken embryos with the USU-BE-Seraing/2017 strain using the allantoic route. Bars indicate means ± standard deviation; *n* = 5 per condition; “*” indicates a *p*-value < 0.05.

On day 5 pi, impaired growth (*p* = 0.002) was detected in the infected embryos compared to controls (Figure 6). The pathomorphological analysis revealed cutaneous hemorrhage without specific microscopic findings, except for the CAM, where cell necrosis and inflammation were marked.

Varying amounts of viral antigens were demonstrated by IHC in the different tissues mentioned earlier, but also in the eye (retina), skin (epidermis and feather follicle pulp), and intestine (Figure 4D–G). USUV-antigen staining in the muscle bundles of the head, trunk, legs, and wings was mild but reproducible in the majority of the infected embryos. No USUV antigens were detected in the brain, kidney, or lung at any time of infection with this viral strain. 

**Figure 7:**
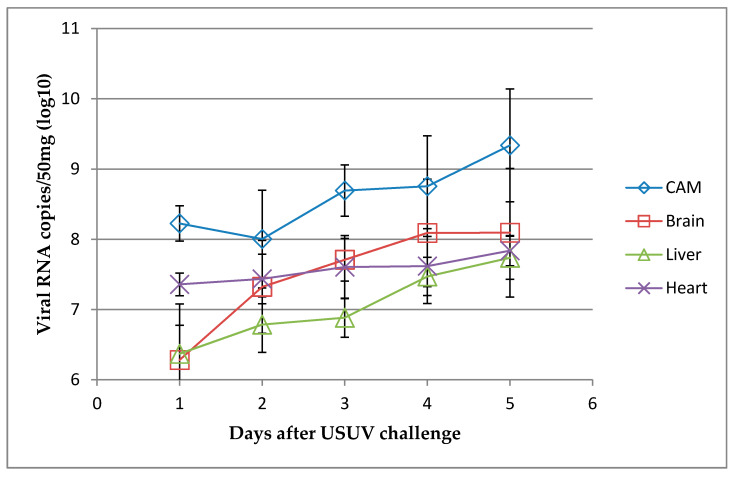
Usutu virus RNA loads detected by RT-qPCR in the brain, heart, liver, and chorioallantoic membrane (CAM) samples of chicken embryos inoculated with USU-BE-Seraing/2017 strain (10^5^ TCID_50_) via the allantoic route. The data show the mean log 10 viral RNA copies/mL ± standard deviation. *n* = 5 per tissue per day of infection.

The CAM, brain, heart, and liver samples all tested positive by USUV-specific RT-qPCR during the infection (Figure 7). A higher viral RNA load was found in the CAM compared to the other three tested tissues (*p* < 0.001). The heart and brain ranked second (*p* = 0.606), with higher amounts of RNA compared to those detected in the liver (*p* < 0.001 and *p* = 0.002, respectively). 

### 3.2. Virulence of other USUV Strains In Ovo 

**Figure 8:**
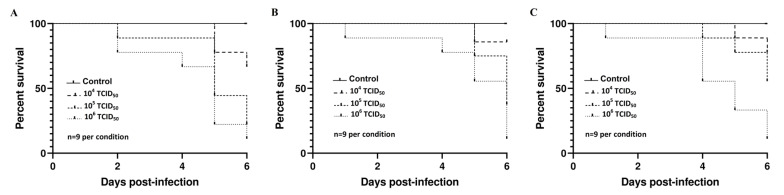
Kaplan–Meier survival curves for chicken embryos inoculated with three different doses of (**A**) Vienna 2001, (**B**) UR-10-Tm, and (**C**) USU-BE-Grivegnee/2017 Usutu virus strains using the allantoic route.

Kaplan–Meier survival curves (Figure 8) revealed dose-dependent mortalities by both the log-rank (Mantel-Cox) and the Gehan-Breslow Wilcoxon tests following infection with USU-BE-Grivegnee/2017 (*χ*^2^ = 11.06 and *p* = 0.004), Vienna 2001 (*χ*^2^ = 7.994, *p* = 0.0184, and *χ*^2^ = 7.7, *p* = 0.0204) and UR-10-Tm (*χ*^2^ = 7.919, *p* = 0.0191, and *χ*^2^ = 7.15, *p* = 0.028) strains. 

No statistical differences were found in the embryonic mortality rates induced by the four USUV strains (Table 1). Similar findings were further observed with European 3 lineage strains USU-BE-Villers aux Tours/2017 (*Genbank*: MK230890, passage 5) and USU-BE-Richelle/2017 (*Genbank*: MK230893, passage 5) [37] (data not shown). Moreover, no lethal effect was observed with doses of less than 10^4^ TCID_50_ using all USUV available in our laboratory (data not shown).

Gross and microscopic lesions, as well as IHC results, were similar to those observed after infection with USU-BE-Seraing/2017 strain, with some new sites of virus replication. Embryos that died on day 5 pi with USU-BE-Grivegnee/2017 and UR-10-Tm strains presented few antigen-positive cells in the brain (Figure 4H). An embryo infected with USU-BE-Grivegnee/2017 strain showed abundant viral antigens in the pituitary gland on day 6 pi (Figure 4I). An overview of the IHC findings using USUV strains is given in Table 2. As for the USU-BE-Seraing/2017 strain, infectious viruses from the AFs and liver tissues of the dead embryos infected with the three USUV strains used in this study were successfully isolated on Vero cell cultures.

### 3.3. Characterization of USUV Strains Growth Kinetics in Chorioallantoic Membrane Cells

**Figure 9:**
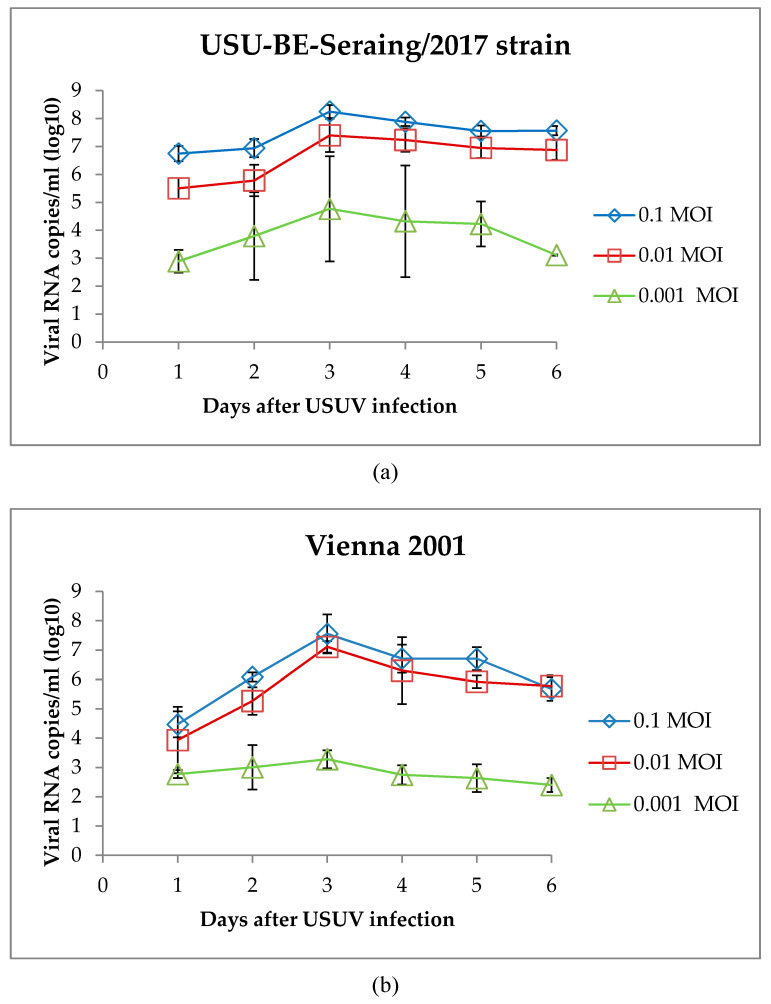

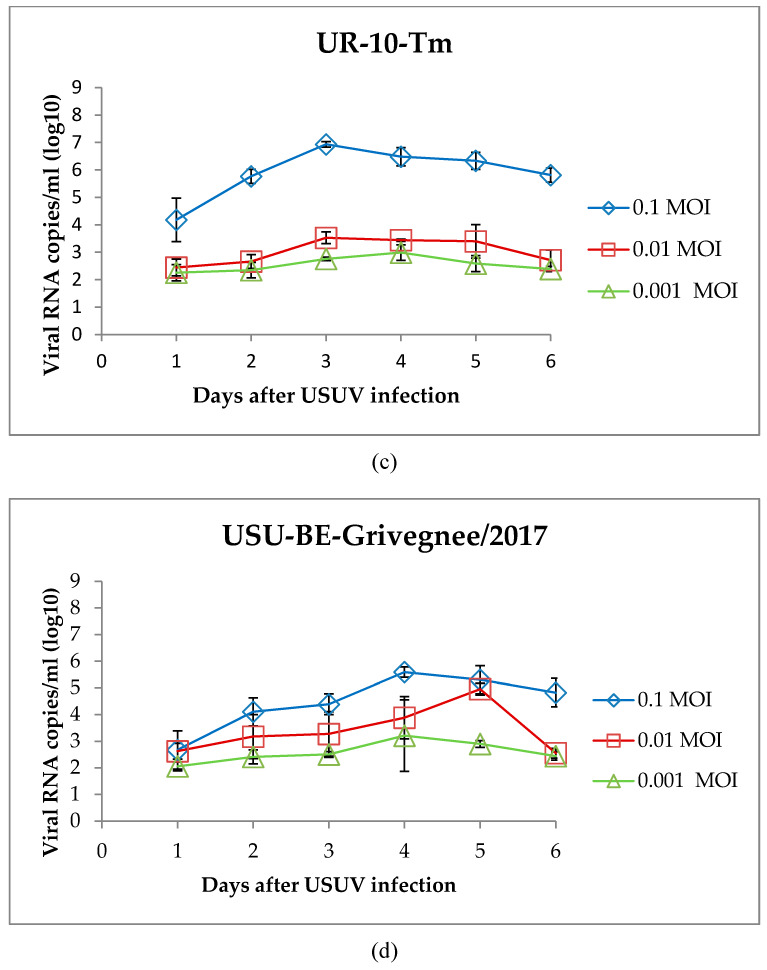
Viral RNA loads in the supernatants of primary cultures of chorioallantoic membrane (CAM) cells infected with different USUV strains (**a**) USU-BE-Seraing/2017 (**b**) Vienna 2001, (**c**) UR-10-Tm, and (**d**) USU-BE-Grivegnee/2017, as determined by RT-qPCR. CAM cells were infected with USUV at MOIs of 0.1, 0.01, and 0.001. Data are representative of three wells per day for each MOI, each performed in duplicate (error bars represent standard deviations).

The RT-qPCR quantification of the USUV genome in the supernatant of CAM cells infected with different USUV strains showed significant variation in viral load according to both MOI (*p* = 0.0004 for USU-BE-Grivegnee/2017 and *p* < 0.0001 for the other strains) and strain. The USU-BE-Seraing/2017 strain produced the highest amounts of viral RNA at all MOI (the difference between Vienna 2001 strain *p* = 0.007, the difference between UR-10-Tm strain and USU-BE-Grivegnee/2017 strain *p* < 0.0001), up to 8.25 log10 VRC/mL with an MOI of 0.1 on day 3 pi (Figure 9a). The Vienna 2001 USUV strain ranked second in terms of RNA amplification in CAM cells (the difference with UR-10-Tm strain *p* = 0.007 and with USU-BE-Grivegnee/2017 strain *p* < 0.0001), followed by USU-BE-Grivegnee/2017 and UR-10-Tm strains, which resulted in similar virus amounts (*p* = 0.279) (Figure 9b–d). Increases of 2- to 70-fold in the RNA loads of USU-BE-Seraing/2017, Vienna 2001 and UR-10-Tm strains were found after the first 72 h for all the MOI tested (Figure 9). On day 4 pi, a drop in VRC was concomitant with massive lysis of CAM cells (not shown). USU-BE-Grivegnee/2017 strain production in CAM cells peaked on day 4 pi with MOIs of 0.1 and 0.001 and on day 5 pi with an MOI of 0.01 (Figure 9d).

**Figure 10:**
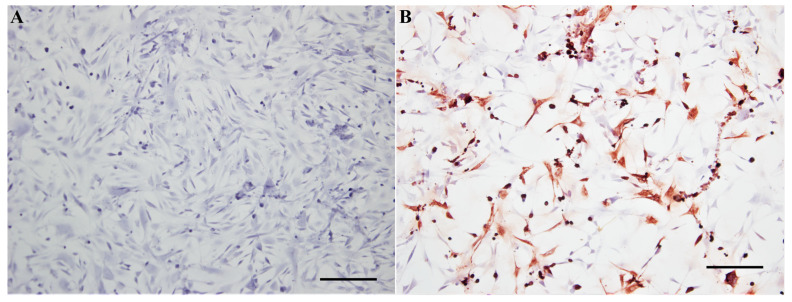
Immunohistochemical staining of USUV antigens performed on chicken chorioallantoic membrane cells. (**A**) Mock-inoculated cells; (**B**) USUV-infected cells. Mayer hematoxylin counterstain. Scale bars = 50 µm.

At the end of the experiment, CPEs were markedly pronounced in the wells infected with MOIs of 0.1 and 0.01 (not shown). The CPEs were characterized by the appearance of rounded, retractile cells followed by cellular death and destruction of the cell monolayer. Abundant antigen signals were seen in the cells remaining in the bottom of the wells, as seen by IHC staining (Figure 10).

## 4. Discussion

In this report, we showed that all four USUV strains injected at high doses in the ECE via the allantoic route successfully replicated in the AF and caused deaths to chicken embryos. These results were in contradiction with three previous studies that inoculated USUV to ECE. In the study carried by Segura et al. [32], the authors infected 10-day-old ECE with high doses (10^4^, 10^5^, or 10^6^ Plaque-Forming Units PFU) of USUV strain V18 (*Genbank*: KJ438730, lineage 3) via the allantoic route. Only low USUV titers were detected in the AFs from 14% of the eggs, and the chicken embryos developed normally [32]. In the study by Bakonyi et al. [31], Vienna 2001 USUV strain was injected into the allantoic sac of 10-day-old ECE at a high dose (6 × 10^5^ TCID_50_). The infected chicken embryos did not show death or lesions after four days of incubation and were negative according to IHC [31]. In contrast, the same strain in our study induced mortality in one embryo at a dose of 10^5^ TCID_50_ and in three out of nine embryos at a dose of 10^6^ TCID_50_ after four days of infection. In our hands, both live and dead embryos at this stage presented pathomorphological changes in the CAM and virus antigens in many tissues (typically in the CAM and skeletal muscle) that were highly indicative of USUV infection (data not shown). In the study by Bakonyi et al. [31], the original USUV isolate (before passaging) and USUV passaged twice in Vero cells exhibited negative results. However, the strain we used for ECE inoculation was passaged 17 times in Vero cells, which may have induced specific genomic changes that increased its pathogenicity for ECE. Another possible explanation for the different infection outcomes by this USUV strain is that susceptibility to the virus might be variable according to the chicken breed from which the embryonated eggs were obtained. Indeed, the immune response to a given pathogen can differ according to chicken lines, contributing, at least in a part, to these differences in the infection phenotype. For instance, the innate immune response to Newcastle disease virus infection was shown to be breed-dependent using chicken embryos [40] or hatched chicks [41] as infection models. Evidence of the role of the interferon response in the control of USUV infection was shown using several in vitro [42,43] or murine models [32,44,45,46]; thus, a breed-dependent, innate immune response to USUV could be the underlying mechanism of the selective pathogenicity of USUV to chicken embryos. The immune response of the developing chicken embryo would be an excellent tool to evaluate the still-unexplored avian innate immune mechanisms in response to USUV infection. Likewise, the investigation of line-dependent chicken embryo immune responses would offer valuable answers to the question of the selective pathogenicity of USUV infection among avian species in general.

The lethal effect of USUV was highly linked to the infective dose, as seen with other flaviviruses, such as ZIKV [28], WNV [33], and Japanese encephalitis virus [47], when injected into ECE. No lethal effect was observed with a dose of less than 10^4^ TCID_50_, and USUV poorly replicated in the AFs and embryonic tissues at a dose of 10^3^ TCID_50_ or less (data not shown). Hence, ECE are likely to have limited efficiency for virus isolation from low-concentrated field samples. This may explain why ECE resisted infection by USUV from dead bird samples in the study of Savini et al. [8], contrary to the Vero cells used in the same study. 

In goose embryos, infection with the Vienna 2001 USUV strain did not cause mortality nor significant gross or microscopic lesions [48]. However, USUV replication was detected in the retina, some autonomic ganglia, skeletal muscle, renal tubular cells, and connective tissue cells [48]. In our report, through intra-allantoic injection of high doses of USUV, the infected chicken embryos showed stunted growth and cutaneous hemorrhage, which are common features of infection with some other mosquito-borne epornitic viruses, such as WNV [49] and Tembusu virus [50,51]. Microscopically, focal necrosis and non-suppurative inflammation were the hallmarks of infection in the CAM. High RNA loads and viral antigens were detected in other tissues, such as the brain, heart, and liver. The lack of inflammation in these organs is not yet well understood. This same feature was found after infection of ECE with the Yellow Fever 17DD vaccine virus [30]. Correspondingly, the liver showed very obvious macroscopic lesions and yielded infectious virus detectable by Vero cell culture; yet, no spectacular histopathological changes, lower RNA loads compared to other tissues, and very few positive hepatocytes were detected by IHC. As a possible explanation, some of the viruses revealed by RT-qPCR and Vero cell cultures were possibly simply circulating in the blood [28]. 

The brain and pituitary gland tissues of embryos occasionally showed viral antigens. USUV was shown to infect several murine and human neuronal cells and to replicate in mature human astrocytes more efficiently than ZIKV [52]. The impact of ZIKV on the development of the central nervous system of chicken embryos was already assessed [27,28], and we estimate that our *in ovo* USUV model provides ground for similar studies in the future. 

In our study, viral antigens were detected in the retinas of the chicken embryos on the second and third days of infection, consistent with the presence of viral antigens in the retina of experimentally USUV-infected goose embryos [31] and the dissemination of USUV to the eye demonstrated by RT-qPCR in experimentally infected canaries (*Serinus canaria*) [39]. Visual impairment and ocular lesions were described in naturally WNV-infected raptors [53,54]. Another flavivirus, Bagaza virus (BAGV), was reported to cause blindness and ocular lesions in common pheasants (*Phasianus colchicus*) and partridges (*Alectoris rufa* [55] and *Perdix perdix*) [56]. Further in vivo experiments in avian and murine models would be necessary to characterize the visual disorders potentially induced by USUV infection. Likewise, during embryonic development in chickens, we demonstrated for the first time the possibility of viral replication in feather follicles. This finding was in accordance with the excretion of USUV via the immature feathers of canaries during the early stages of experimental USUV infection [39]. These preliminary observations suggested that feathers may potentially play a role in the spread of the virus. Fully grown feathers from either dead or live birds of all ages and molt cycles could provide a simple method for the detection of WNV infection [57]. Further, the Israel turkey encephalitis virus, a deadly flavivirus for turkeys in Israel, could be amplified from feather pulps; virus detection from such samples was proposed to evaluate the proper administration of live vaccines [58]. More studies are needed to characterize the capacity of USUV to disseminate via the feathers in both naturally and experimentally infected birds [39].

The virus replicated in different regions of the egg, preferentially in the AF and CAM. In the AFs, the significantly higher RNA loads detected during the first four days of infection compared to the first day could indeed rule out a simple detection of remnants of the viral inoculum by RT-qPCR. A peak was found in the RNA loads of the infected embryos on day 3, making it the most suitable day to collect AF for virus amplification. Infectious virus was systematically retrieved from the AFs of dead embryos using Vero cell-culture, further indicating the active replication of the virus in this region of the egg. Higher VRC were found in the AFs from dead embryos than in those from surviving ones, suggesting that higher replication in this site prompts fatal outcomes of USUV infection. The Yellow Fever-17D vaccine is considered to be among the most successful live-attenuated human vaccines and was used to develop other flavivirus vaccines by chimerization [29]. It was obtained by serial passages of the virus in chicken embryo tissues to remove its neurotropic properties [29]. Our ECE model could be beneficial to test the protective effect of vaccine candidates, but its efficiency to amplify virus particles in large amounts as needed for the vaccine industry is questionable due to the high virus input needed to obtain viral replication in the AF. 

Evidence of strong viral replication was seen in the CAM. This result resembled that observed following infection of ECE with WNV [49], but it did not match with that obtained with the Yellow Fever 17DD vaccine virus, which did not replicate in the CAM [30]. Consequently, CAM cells were isolated in vitro and showed susceptibility to USUV infection, as evidenced by the appearance of characteristic CPE and viral RNA production. To our knowledge, goose embryo fibroblasts were the only available in vitro avian model for the study of USUV, until now [31]. Here, we developed the first cellular model from domestic chicken (*Gallus gallus domesticus*) allowing the study of USUV. Virus quantities were directly related to seed virus input, which may limit the cost-effectiveness of this model in vaccine production. The yield of virus per cell [59] should be determined to characterize the production efficiency of this virus using this model. 

Primary chicken CAM cells were used to compare the replicability of multiple phylogenetically distinct USUV strains, and differences in growth kinetics were observed. The USU-BE-Seraing/2017 strain showed the highest viral replication using this model, providing an interesting model for the evaluation of the USUV sensitivity to antivirals, for instance. Whether the passage of virus in CAM cells led to the selection of genetic variants needs to be determined by nucleotide sequence analyses and *in ovo* pathogenicity assessment of CAM cell-derived strains. 

## 5. Conclusions

In conclusion, this report is the first to use ECE and chicken embryo-derived cells as artificial models to study the histopathological lesions and virus tropism involved in the pathogenesis of USUV. Our data suggested that USUV infection in *Gallus gallus domesticus* embryos is systemic and lethal in a dose-dependent manner. The CAM seems to be the main replication site of USUV, with severe histopathological changes and abundant cell staining by IHC. Cells from the CAM were highly permissive to USUV when cultured in vitro. We believe the use of this model, along with ECE, could further foster a significant understanding of the pathogenesis and provide grounds for the development of vaccines against USUV.

## Figures and Tables

**Table 1:** Chicken embryo mortality rates comparison following the infection with three different doses of four Usutu virus strains and using log-rank (Mantel-Cox) and Gehan-Breslow Wilcoxon tests.

Viral Dose (TCID_50_)	Log-Rank (Mantel-Cox)		Gehan–Breslow Wilcoxon	
	*χ* ^2^	*p*	*χ* ^2^	*p*
10^6^	3.846	0.2752	3.537	0.316
10^5^	2.033	0.5655	2.203	0.5113
10^4^	0.03672	0.9981	8.845e^-032^	>0.9999

**Table 2:** Usutu virus antigens distribution in the selected tissues samples of infected chicken embryos, as determined by immunohistochemistry.

Tissue	Infection with USU-BE-Seraing/2017					IHC Findings in Embryos Infected with Other USUV Strains *
	dpi					
	1	2	3	4	5	
**CAM**	-	++	+++	+++	+++	Common to all strains
Brain	-	-	-	-	-	Positive staining when infected with USU-BE-Grivegnee/2017 and UR-10-Tm strains (day 5 pi)
Heart	-	-	+	+	+	Common to all strains
Liver	-	-	+	-	-	Only with USU-BE-Seraing/2017
Skeletal muscle	-	+	+	+	+	Common to all strains
Intestine	-	-	-	-	+	Positive staining with USU-BE-Grivegnee/2017
Eye	-	+	+	-	-	Only with USU-BE-Seraing/2017
Skin and feather follicles	-	-	+	++	+++	Common to all strains

+++: high; ++: moderate; +, low; -: no antigen detected; IHC: immunohistochemistry; c.e: chorionic epithelium; dpi: days post-infection; USUV: Usutu virus. * Data gathered from dead embryos tested during the lethal test with three USUV strains, i.e., USU-BE-Grivegnee/2017, Vienna-2001, and UR-10-Tm.

## Supplementary Materials

The supplementary materials are available online at https://www.mdpi.com/1999-4915/12/5/531/s1.
